# The mechanism of goal-setting participation’s impact on employees’ proactive behavior, moderated mediation role of power distance

**DOI:** 10.1371/journal.pone.0260625

**Published:** 2021-12-15

**Authors:** Sabeeh Pervaiz, Guohao Li, Qi He

**Affiliations:** 1 School of Management, Jiangsu University, Zhenjiang, China; 2 School of Finance and Economics, Jiangsu University, Zhenjiang, China; Rzeszow University of Technology: Politechnika Rzeszowska im Ignacego Lukasiewicza, POLAND

## Abstract

Organizations nowadays are under immense external pressure due to advancements in information technology, making it precarious. It also inserts extra pressure to keep the employees motivated and productive. Therefore, while information technology benefits the organization, it also challenges the organization and employees more. In order to meet these challenges, many organizations have begun to flatten their organizational structures and decentralized their management approaches. This study collected 336 valid questionnaires from 20 service companies. The reliability and validity of the questionnaire were tested. In addition, the exploratory factor analysis and confirmatory factor analysis. Relevant analysis and empirical analysis were also carried out using hierarchical regression. The study finds that (1) Goal-setting participation positively affects employees’ proactive behavior. (2) Perceived insider status plays a mediating role between goal-setting participation and employee proactive behavior. (3) The power distance positively modifies the goal-setting participation in the relationship of employee’s perceived insider status. (4) Power distance positively moderates perceived insider status in the relationship of goal-setting participation on employee proactive behavior through perceived insider status. This research applies goal-setting theory and social cognition theory to build a theoretical framework for the influence mechanism of goal-setting participation on employee’s proactive behavior. Expands the application scope of fundamental theoretical research and improve understanding of the relationship between goal-setting participation and employee’s proactive behavior. The research conclusions help organizations understand the formation mechanism of employees’ proactive behaviors, strengthen the focus on goal-setting participation, and optimize the relationship between leaders and employees.

## Introduction

This rapid development era in information technology brings more intense, complex, and unpredictable competition for the organization, making it hard for the organization to keep the employees engaged and active. Talented and enthusiastic employees are an essential part of any organization, as organizations’ performance depends heavily on them. Unfortunately, very little research has been done on how organizations, via education and training, care for their employees on a larger scale than in the industry. Human capital is typically recognized as the only resource to be enhanced continually. In long-term efforts to increase the level of organizations, it may be considered significant [1]. The realization of the learning organization idea became relevant when businesses used staff education and training to boost growth, competitiveness, and adaptability to external changes. The establishment of a learning organization has become a critical component of long-term corporate development, assisting in acquiring and maintaining a competitive edge [2].

It is vital to look for the antecedents that can keep employees motivated and beneficial. The previously positive effect of employees’ proactive behavior on the organization concluded that employees could actively provide job suggestions and feedback to overcome difficulties and improve their performance [3]. Through active collection and feedback, new employees can clarify their job responsibilities, information, role imitation, and role positioning in the work process more quickly [4]. Employees’ active participation in daily social interactions can build good subordinate relationships and create a decent working atmosphere. However, modernization has brought many changes to the work setting now. Organizations often make decisions that are not user-friendly; thus, employees feel inactive when they are imposed to follow the supervisor’s orders [5]. Employee behavior is influenced by various internal (family and personal life, supervisors’ behavioral integrity, trust) and external (work culture, job responsibility, effective communication, work relationships) variables [6]. Other modern factors that may influence employee behavior are the effectiveness of remuneration regulation, identifying "bottlenecks," substantiating socially responsible programs. Identifying priority areas and developing management, Decisions, and monitoring the implementation of planned regulatory measures in remuneration [7]. Any organization’s productivity is heavily dependent on the commitment and dedication of its employees. Therefore, how to effectively stimulate employee’s proactive behavior has been the main idea of this paper.

Proactive employees are an essential asset for any organization, and retaining such employees has always been a big concern for organizations. Because of the information asymmetry between job seekers and employers, potential employees are choosing insufficient educational topics and, as a result, investing in insufficient competencies [8]. Highly qualified people are likely to have relevant, on-demand skills, become more easily integrated into society because they have improved foreign linguistic and cultural knowledge; have better access to world information sources through social and professional networking services. As a result, middle-income nations, where people can afford to go, have greater emigration rates. The entry of highly qualified migrants increases the destination country’s human capital and creates a suitable competitive environment for local inhabitants, encouraging training and acquiring new information and skills [9].

Many scholars have contributed a lot through research. However, Previous research has paid more attention to the antecedent variables of employees’ proactive behavior, such as personality traits, positive emotions, and work motivations [10–12]. Most scholars do not consider the effect of goal-setting and decision-making participation on employees’ positive psychological state, which may strengthen employee’s proactive behavior. Setting goals in our daily life can keep the employees engaged and active in their approach. According to the goal management theory proposed by Peter Drucker, goals have a significant incentive impact on employees’ job motivation, attitude, and behavior [13]. At the same time, goal-setting can become a vital driving force for employees to engage in work that promotes organizational and personal goals. A goal can have several types, whereas goal-setting participation is a particular type of employee decision-making [14]. Participating in goal-setting can affect the individual’s importance evaluation and accessibility of the goal, affecting their sense of control and acceptance of the goal achievement [15]. It enables employees to understand the organizational goals clearly and remain aligned with organizational values. This stimulates a series of positive actions to optimize their work systems, achieve common organizational development goals, and generate insider identity [16]. Goal-setting is how employees can participate in the management and create an organizational atmosphere of trust and respect.

The employees’ willingness to participate in goal-setting and affect their attitudes and behaviors depends on the power distance between leaders and employees. Employees with lower power distance have a more substantial need to be respected. In comparison, Employees with higher power distance have a higher acceptance of unequal power distribution [17]. If employees are treated accordingly, It makes employees more likely to feel respect, recognition, and support from the organization. An essential part of the organization is strengthening employees’ internal identity perception and consolidating insiders’ identities [18]. Therefore, employees will take a series of actions to promote organizational development. Introducing the variable of power distance as a moderator will help interpret the specific goal-setting participation mechanism in the organizational context. It will also help understand the influence of different power distance levels in goal-setting participation affecting perceived insider status.

Locke first proposed Goal-Setting Theory in 1967. The theory believes that setting appropriate goals can enable individuals to achieve goals and motivate employees. In setting goals, employee participation and employees’ feedback on completing goals are key elements motivating employees. They allow employees to clarify the difficulty and clarity of goals and increase employees’ grasp and recognition. In achieving goals, achievement needs will be transformed into intrinsic motivation, making people work hard to achieve goals. In this process, individuals will constantly compare their behavioral results with the established goals. They will adjust and improve their efforts in real-time to ensure the realization of the goals [14]. Clarity and difficulty are the two fundamental attributes of a goal. The effect of target motivation is affected by these two attributes and the surrounding environment. Yukl and Latham proposed a comprehensive model of Goal-setting. When setting goals, employees should be involved in the organization’s goal-setting. Factors such as individual differences among employees should be considered [19]. Locke and Latham [20] further conducted empirical research on survey subjects in different industries and occupations. They found that clear and challenging goals can improve employees’ work performance. Goal-setting theory has been widely used to improve the work performance of employees. Goal-setting participation can explain a series of work attitudes and behaviors of employees. Goal-setting participation can make employees clarify the difficulty and feasibility of goals, increase their autonomy, feel the organization’s respect, significantly improve employees’ enthusiasm at work, and indirectly affect employees’ behavior.

American psychologist Bandura proposed social cognitive theory in 1986. This theory mainly explores the relationship between the environment, cognition, and behavior of the individual. Previous studies have mostly applied social cognition theory to study the impact of organizational management on individual anti-productive behaviors and advocacy behaviors [21]. Compeau et al. [22] applied social cognition theory to establish an individual’s social cognition mechanism in "environment-cognition-behavior." Employees process according to the information obtained from the environment and use this to construct self-awareness and behavior to keep consistent with the external environment. Goal-setting participation is a specific work situation, which is adequate to become an employee’s specific work environment affecting employees’ self-perception and behavior. Therefore, it can be combined with social cognitive mechanisms to study the goal setup mechanism to influence participation on employees’ proactive behavior.

Hence this study expands research of goal-setting participation in several respects. First, this research explored the influence mechanism of goal-setting participation on employees’ proactive behavior in the presence of goal-setting theory. Many researchers widely accepted and explored this theory in similar contexts [23, 24]. It is reasonable to include the goal-setting theory and social cognitive theory in the study of goal-setting participation on employee proactive behavior. Secondly, this research examined the mediating effect of perceived insider status in this research setting, which will help the organizations better understand employees’ perceptions and outcomes. Third, the variable of power distance is introduced as a moderator between goal-setting participation and perceived insider status. This study hypothesizes that power distance can play a vital role in the overall relation. It will help the managers to understand their employees and design the goals accordingly. Hence, this study expands the theoretical basis’s scope. Additionally, it enriches the theoretical literature and empirical research on goal-setting participation and the antecedent variables of employees’ proactive behavior. Most importantly, this research provided some enlightenment to the management practices, such as encouraging employees to participate in the management, optimizing the relationship between leaders and employees. It will help create a harmonious working environment, improve the management levels, and promote its healthy development in the long term.

## Hypotheses development

### Participation in goal-setting and proactive behavior

Nowadays, organizations are more concerned about employee involvement in setting up organizational goals. Internal difficulties, such as inefficient hiring, continuous improvement, job structure, and motivation, are the primary challenges in modern personnel management at such companies [25]. For organizations that desire talent onsite rather than remote contract employees, a scarcity of experienced applicants in the area might be a significant issue. SME’s, which, in principle, appear to be the most adaptable for use with modern personnel levers, have proven inadequately intimate [26]. Employees have become more educated and have high negotiation skills [27]. Managers also support this concept of employee involvement in setting up their goals, which gives employees motivation and managers the supremacy to motivate and direct their employees to achieve those goals. According to Erez et al. [28], Participation in goal-setting refers to employee involvement in setting up the goals. Participation in goal-setting makes the goals more acceptable and leads to more involvement from employees. Organizations and managers allow employees to work together to set up the organization’s goals. It helps the employee with more involvement and proactive behavior [29]. In setting goals, employees can have intrinsic motivation and desire to obtain them, positively affecting organizations in the longer run. Employees can also make observations and recommendations about the content and standards

Many scholars have previously explored goal-setting participation to increase the individual’s understanding of completing the organization’s goals, increasing the goals’ acceptance, and improving the employee’s goal commitment level [30–32]. Employees’ trust in managers is enhanced when they have a reduced power distance [33], clarifies the guidelines in goal completion [34], and improves employees’ internal motivation [35]. The employee’s inner motivation determines the employee’s work attitude and behavior. In contrast, extrinsic motivation plays a vital role in decreasing the power distance as an employee feels connected and part of the organization.

This study proposed the following views. Based on the previous discussion and literature review, goal-setting participation can increase employees’ proactive behavior [23]. This assumption supported the goal-setting theory; participation in goal-setting can increase the individual’s understanding of achieving its goals [36]. However, it also increases the acceptance of goals and improves the employee’s goal commitment [37]. Studies have shown that employees’ independent decision-making power promotes trust and intrinsic motivation through participation. Building a positive emotions model to promote employee initiative can play a driving role in increasing employee initiative behavior [38]. Participating in the goal-setting process can increase the possibility of achieving the goal, as employees will control the goal’s difficulty within the scope of their ability [39].

Secondly, Goal-setting involvement increases an employee’s sense of self-efficacy. Çetin & Aşkun [40] research suggests that employees’ self-efficacy improves employees’ internal work motivation and promotes proactive behavior. This concept is well supported in the light of the Social learning theory Bandura [41]. It is defined as”*people’s beliefs about their capabilities to produce designated levels of performance that exercise their influence over events that affect their lives*.” Personal belief in the capabilities can drive a person to set a goal of high standards. Goal-setting attaches to a person’s belief or confidence in achieving the task. If a person has strong self-efficacy, they will set higher standards goals and have a firmer approach in achieving goals [42]. Similarly, Shoaib & Kohli [24] suggested that managers focus on the employee’s contribution and participation in the goal-setting process only when positively influencing the employee’s initiative.

Furthermore, this study proposed that communication is the key to any good relationship. According to Grunig [43], businesses should treat employee relations in the same way they treat other critical stakeholders: Grunig further said that good employee communications might lead to better employee relationships and more supportive employee actions toward businesses. Employees who receive favorable feedback on their performance are more likely to preserve reliable connections with their employers. Good communication between the company and employees and among coworkers is the foundation of long-term engagement [44]. In addition, the communication environment and system of an organization are significant contextual factors that influence involvement. Translucent organizational communication is indeed an organization’s premeditated dissemination of information in combination with its active involvement in information acquisition and distribution to hold companies accountable for business legislation and processes in a genuine, substantial, and comprehensive way [45]. Both controls and communication are key elements to create a management system that can increase innovation and market performance standards among small and medium-sized enterprises. In each small and medium-sized business, they both participate in the distinctive culture of a business [46].

Communication between employees and managers increases the employee’s understanding of the goals and minimizes the power distance [47]. Employees feel more connected, motivated, and attached to the organization when they believe they hear their voice. David [48] found that employee participation management can help employees understand their roles better and recognize the development strategy. Employees perceive a high degree of trust in the organization, generate a sense of accomplishment, and belong to the organization [49]. This sense of belonging and achievement drives employees to generate positive emotions, improve organizational commitment, optimistic attitude, and proactive behaviors. Many researchers in the West have also researched different types of employees and industries and reached the same conclusions [50, 51]. Employees working in different industries have the exact needs and desire to be recognized by their organization and managers. Hence we can expect participation in goal-setting to have a positive relationship with employee active behavior.

*Hypothesis 1: Participation in goal-setting positively affects employees’ proactive behavior*.

### The mediating role of perceived insider status

Perceived insider status (PIS) relates to how employees believe themselves as part of an organization [52]. When employees feel more connected to an organization, they will exhibit positive behavior towards the organization. Previous researchers have studied perceived insider status with two different approaches. According to inducement contribution theory, Employees perceive themselves as an insider of the organization and an integral part of the organization setting. They believe the organization has provided them personal space and acceptance [16]. Whereas organizational socialization theory relates that employees feel connected to an organization and help new employees gather essential information about the company [53]. Studies showed that employees who feel connected and valued at the start of their jobs have long tenures and productivity [54, 55].

Participation in goal-setting is a specific work situation that creates a work environment that can determine the organization’s feelings. The organization is willing to empower employees to participate in the organization’s goal-setting and employees have the right to participate and speak [56]. This way of empowering management sends a signal to employees that the organization recognizes its capabilities. Managers and employees have formed a mutual sharing, mutual respect, mutual tolerance of the common work model [57]. Research conducted by Ding [52] also discussed the positive relationship between participation in goal-setting and perceived insider status. Participation in goal-setting allows employees to feel the organization’s recognition of their capabilities. It creates an atmosphere of trust in the organization [58], respect of the organization [48], and an increased sense of belonging [18].

Employees with strong insider status “insiders” of the organization have a stronger sense of emotional connection and belief, resulting in spontaneous, forward-looking, and transformative initiatives [16]. This results in many behaviors conducive to organizational development, such as organizational citizenship behavior. Wang [18] proposed that employees with strong insider identity awareness prioritize the organization’s interests over personal interests and are more motivated to work. Employees will generally think about organizational-related issues from a long-term perspective of insider behavior. They will actively seek and share solutions and be better at overcoming obstacles at work. Favero [59] suggests that strong insider perception makes employees more willing to take on challenging tasks. They will produce innovative behaviors that are conducive to the development of the organization. Based on the previous research, we propose that perceived insider status plays a “bridge” role between goal-setting participation and employee proactive behavior. Based on this self-awareness, employees will think that they have common interests with the organization.

This research also supports the previous concept of Goal-setting involvement, making employees aware that the organization’s goals are closely linked to their goals. It creates a sense of self-awareness that they share the same interests as the organization [60]. When employees participate in the organization’s goal-setting process, the employees will provide personal opinions and suggestions on the difficulty and accuracy of the goals under their circumstances [61]. Nowadays, employees are more concerned with the goals setting accuracy and difficulty. They do not encourage the passive approach from the organization in setting up the goals. Employees will have a clearer understanding of the organization’s goals and increased acceptance of goals [59]. When the employees and the organization’s goals are consistent, the value of the employee’s organizational relationship is improved, and employees will consider themselves part of the organization. Based on the above discussion, we can hypothesize the positive relationship between participation in goal-setting and perceived insider status.

*Hypothesis 2*: *Perceived insider status mediates the relationship between goal-setting participation and employee proactive behavior*.

### The moderating role of power distance

With a positive nature of participation in goal-setting on employee proactive behavior, Many researchers have introduced the variables such as autonomous motivation [62] and employee trust [63] overall effect to measure this relationship. This section of the paper introduces power distance as a moderator. Power distance refers to the distribution of power in an organization and its acceptance from employees [33]. Employees can have a dual approach to this power distance. Employees who believe power distance should exist in the organization are referred to as high power distance employees. Whereas some employees support, managers having less control over their subordinates are low power distance employees [64]. Employees with high power distance tend to obey the leadership’s work pattern. In contrast, employees with low power distance tend to work in a decentralized way. This study discusses the power distance in a dual approach.

First, Employees who believe that the manager should hold power over their subordinates support the idea of having a formal relationship at work. Such employees believe attitudes and performances are not dependent on power distance [17]. Employees at a high power distance often think that leadership and employees are not equal, and employees are bound to comply with managers. Research by Wang and Guan [64] also supports that such employees will accept the organization’s centralized management and are inclined to follow the authority. Employees believe that top management is responsible for the decision-making process and rarely question their decisions. In organizations requiring employees to participate in the organization’s goal-setting, employees with high power distances will choose conservative strategies [65]. “Especially in the process of participating in goal-setting, employees with high power distances are less willing to participate in the organization’s goals. if there is a high level of uncertainty about a factor in the goal-setting process.”

On the other hand, some employees believe that their organization must include them in the decision-making process or feel left out. Such employees desire low power distance [66]. Employees with low power distance levels are willing to participate in the organization’s goal-setting. Studies have shown that employees with low power distance display negative behaviors when they are not included in goal-setting [67]. Research by Wang and Guan [64] states that authoritarian leaders are expected to affect low power distance employees negatively. This study argues that low power distance need is related to low perceived insider status. With less power distance, employees will have more access to organizational matters and increased insider identity. Hence it propose that power distance act as a moderator in the relation of participation in goal-setting on perceived insider status.

*Hypothesis 3*: *Power distance will moderate the relationship between participation in goal-setting on perceived insider status*. *High power distance will result in high perceived insider status*, *and lower power distance will have lower perceived insider status*.

### The moderated mediation role of power distance

As per the previous research summary and theoretical analysis, there is a correlation between goal-setting participation and employee’s perception of insider identity. Also, there is a correlation between the perception of insider’s identity and employees’ proactive behavior. However, the difference in power distance may affect the degree of employee participation in goal-setting. Power distance is an organization, group, staff, and other psychological perception of uneven power distribution. The distribution of power imbalance is more likely to be accepted in higher power distance organizations.

Previous studies have not directly tested the moderating effect of power distance between participation in goal-setting and perception of internal identity. In addition, previous studies have mainly studied the impact on management practices from the differences in the power distance of employees themselves and rarely comprehensively considered the level of power distance between organizations, managers, and employees [68]. There is not much research on the participation of the three power distance levels brought to management practice. Consequently, the level of power distance between organizations, managers, employees and the difference in power distance between the three factors that need to be considered affect employees’ participation in goal-setting. Managers with different power distances will have different management styles and different perceptions of employee behaviors and ways of responding to employee behaviors [69]. Employees with different power distance levels are willing to participate in the organization’s goal-setting to various degrees. Managers with different power levels are willing to let employees participate in the organization’s goal-setting [70]. According to the theory of social cognition, employees’ internal perception of identity is also unique [71].

Most previous research has used power distance as a moderating variable due to its controlling nature. Many studies have been conducted on the moderating effect of power distance at the individual level among various variables [67, 72] For example, the moderating effect of power distance between different types of leadership styles [73], management styles and employee behaviors [68], superior-subordinate relationships [74], and organizational performance [64]. The power distance of employees negatively regulates shared leadership and employee admonition behavior [65]. Employees with high power distance will reduce conflicts with leaders and other members in the work process and negatively regulates the relationship between moral leaders and subordinates. Virtue leaders create a respected and trusted working atmosphere for employees; hence employees with low power distance have stronger motivation to interact with leaders. Therefore, It is easy to establish a good relationship between superiors and subordinates. The power distance of the group positively regulates the relationship between the management method of the organization’s differentiated authorization, employee fairness perception, and employee performance [47]. The group with high power distance believes that the differentiated authorization is the organization’s comprehensive consideration of employee differences and the choices made can improve the performance of the organization.

In summary, the power distance at all levels has a range of effects on employees’ proactive behavior. In a country with a high power distance like China, the influence of the different power distance levels of the organizations or individuals studied has essential theoretical and practical significance on employee behavior. Therefore, power distance moderates the mediating variable of perceived insider status variable to explore the influence mechanism of goal-setting participation on employees’ proactive behavior.

*Hypothesis 4: Power distance will moderate the mediating role of perceived insider status between Participation in goal-setting and Proactive behavior. While Power distance is high, the mediating effect will be enhanced; When Power distance is low, the mediating effect is weakened*.

**Fig 1:**
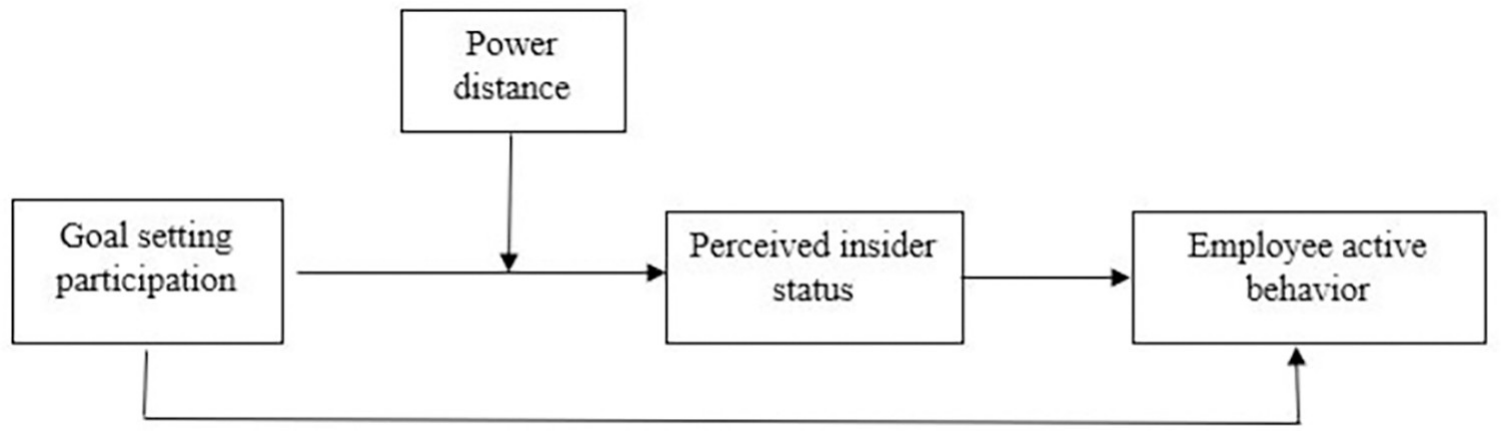
Theoretical model.

The theoretical model can be seen in Fig 1.

### Methodology

The service industry is one of the fast-growing industries in China and explored by many researchers. The questionnaire was designed on a weblink and circulated to HR managers of 20 service companies (including 7 hospitals, 4 logistic services, 5 travel companies, and 4 telecom companies). 20 questionnaires were sent to respective HR managers for dissemination among employees. Since the respondents had to fill the questionnaire in three phases with time intervals, a code was assigned to each respondent.

The first phase of data collection measured goal-setting participation, power distance, and demographic information initiated in January 2020. 440 total questionnaires were distributed, and 386 were received. Upon further assessing the returned questionnaires, incomplete and influenced questionnaires were deleted in every phase. Therefore 380 valid questionnaires were recovered during phase one.

The second phase of data collection was carried out to collect data on perceived insider status in April 2020. 380 questionnaires were returned to the respective employees, and 364 were received. The valid questionnaire during this phase was reported as 362. The third phase of data collection commenced in June 2020. 362 questionnaires were sent back to measure employees’ proactive behavior, and 347 responses were received. Upon the final validation of questionnaires, 336 valid data sample was obtained with a response rate of 76.36%. All the research processes were rigorously monitored and standardized. Participants were asked to complete the survey items uniformly within the prescribed time limit, and the purpose of the research was conveyed to participants. Therefore, the objectivity, confidentiality, and authenticity of the collected data were ensured to a certain extent.

The distribution of men and women is relatively balanced, of which 59.83% are male and 40.17% are female. In terms of age, 22.62% are under 25,51.19% are 26–35, 17.56% are 36–45, and 8.63% are over 46. In terms of education level, the proportion of undergraduates is 46.72% at the maximum, and 14.29% of those with a junior college education or below. In terms of tenure, 27.08% of respondents had less than 5 years spent in the company, 24.40% had spent 6–10, 24.40% had spent 11–16 years, where the most number of respondents reported in tenure of 17–25 years with 35.42%,and above 26 years reported as 3.57%.

### Ethical issues

This study was carried out in accordance with the recommendations of the Ethical Principles of Psychologists and Code of Conduct by the American Psychological Association (APA). The research was granted access by the ethics committee of the School of Management, Jiangsu University, Zhenjiang, the People’s Republic of China, as a part of Ph.D. Dissertation. The Industrial linkages between the university and service industries supported getting the approvals from participating companies’ employees council. The participating companies’ HR departments were informed, and all the participants provided the written consent. The research was conducted in compliance with the Declaration of Helsinki.

### Scales selection

#### Goal-setting participation

The goal-setting participation is based on a scale developed by Sholihin et al. [75] with a Cronbach α reliability coefficient of 0.719. The scale contains 2 items. Among that is, "the organization allows employees to participate in setting related work goals, and employees participate in work related to themselves."

#### Employee proactive behavior

The employee proactive behavior scale was developed by Frese et al. [76] and the Cronbach α reliability coefficient is 0.640. The scale has a total of 7 items. However, confirmatory and exploratory tests found that deleting 3 of these items can improve the questionnaire’s validity. Therefore, the scale contains 4 items, such as "Every time a problem occurs, the employee will immediately find a solution; usually, you will do more than required," and so on.

#### Power distance

Power distance draws on a scale compiled by Culpepper & Watts [77]. This scale was obtained based on the research of Hofstede. The Cronbach α reliability coefficient is 0.919. The scale has a total of 8 items, such as "corporate rules cannot be broken, even if employees believe that changes are beneficial to the company’s interests on work-related matters. Managers often expect subordinates to obey unconditionally."

#### Perceived insider status

Stamper & Masterson [78] developed the scale of perceived insider status. The Cronbach α reliability coefficient is 0.84. The scale includes 5 items, as "employees feel that they are insiders of the organization, and the organization often makes employees feel that they have not been left out."

#### Control variables

This study considers that demographic variables may affect the relationship between goal-setting participation, power distance, perceived insider status, and employee active behavior variables, so it controls the respondents’ gender, age, education, and tenure.

## Results

### Reliability and validity test

This study used KMO and Bartlett’s spheres to examine the factor analysis suitability. The KMO coefficient is 0.883, which is greater than 0.7, and the significance of Bartlett’s sphericity test is 0.000, which is less than 0.001.

Since the data was obtained through self-assessment by the respondents, in order to reduce common method bias, this study performed Harman’s data on the four variables of goal-setting participation, power distance, perceived insider status, and employee proactive behavior. There are four factors with eigenvalues greater than 1, consistent with the number of variables set in the study. The factor loading matrix extracts four principal components consistent with the study setting [79]. The cumulative variance contribution rate is 66.863%, within the acceptable range [80]. The first-factor variation contribution rate is 33.848%, which is lower than the empirical standard threshold of 40%. This shows no serious common method deviation between these four variables.

This study used AMOS 21.0 to conduct a confirmatory factor analysis of the survey data. This verifies the matching of the data with the model and the validity of the data, as shown in Table 1. The research results show that the four-factor model has the best fit (χ2 / df = 3.198, RMSEA = 0.071, CFI = 0.980, TLI = 0.964). It can be seen that goal-setting participation, perceived insider status, power distance, and employee proactive behavior have good reliability and discriminative validity. The data obtained from the questionnaire is valid.

**Table 1:** Confirmatory factor analysis.

Model	Variable	χ2	df	χ2/df	CFI	TLI	RMSEA
Four-factor model	PGS, PIS, EPB, PD	649.117	203	3.198	.980	.964	.071
Three-factor model	PIGS+PD,PIS,EPB	800.271	206	3.885	.840	.821	.093
Two-factor model	PIGS+PD+PIS,EPB	1403.937	208	6.750	.679	.643	.131
One-factor model	PIGS+PD+PIS+EPB	1770.868	209	8.473	.581	.537	.149

Note PGS; participation in goal setting, PIS; perceived insider status, EPB; active employee behavior, PD; power distance

This research conducted the Fornell Larcker test to check the discriminant validity. The results showed that AVE’s square root values of goal-setting participation (0.646), perceived insider status (0.669), employee proactive behavior (0.665), and power distance (0.707) presented in Table 2 are more significant than other correlation coefficients in the same rows and columns, stating that the variables have good discriminant validity.

**Table 2:** Descriptive statistics and correlation analysis.

	mean	sd	1	2	3	4	5	6	7	8
**1 Gender**	1.5268	.50003								
**2 Age**	2.0982	.83176	-.096							
**3 Education**	1.9613	.49625	.034	-.012						
**4 Tenure**	2.6042	1.25109	.091	.241***	.033					
**5 PGS**	3.4494	.85891	.010	-.058	-.075	.005	**0.646**			
**6 PIS**	3.4732	.80134	-.009	.006	.006	-.025	.378***	**0.669**		
**7 EPB**	3.4769	.75138	-.059	.238***	-.028	-.054	.382***	.483***	**0.665**	
**8 PD**	3.3720	.80616	-.036	-.004	-.052	-.031	.306***	.429***	.396***	**0.707**

Note

1) *** p<0.001, ** p<0.01, * p<0.05

2) The numbers on the diagonal are $\sqrt{AVE}$.

3) PGS; participation in goal setting, PIS; perceived insider status, EPB; active employee behavior, PD; power distance

### Descriptive statistics and correlation analysis

The mean, standard deviation and correlation coefficient of these four variables are shown in Table 2. There was a significant positive correlation between goal-setting participation and employee proactive behavior (r = 0.382, p <0.001). Goal-setting participation was significantly positively correlated with employee’s perceived insider status (r = 0.378, p <0.001). Perceived insider status was significantly positively correlated with employee active behavior (r = 0.483, p <0.001). Goal-setting participation has a positive relationship with employees’ perception of insider identity and employee’s proactive behavior. Whereas perceived insider status also positively relates to employee’s proactive behavior, providing a base for hypothesis 1. The correlation coefficients between the various variables are lower than 0.75, indicating no multicollinearity between the variables, supporting the subsequent testing and regression analysis.

### Test of mediating role of perceived insider status

Goal-setting participates in the primary effect test that affects employees’ proactive behavior. Among all the main effects analysis models, gender, age, education, and tenure were used as control variables for the study. The test results are shown in Table 3. It can be seen from Model 4 that goal-setting participation has a significant positive impact on employee proactive behavior (β = 0.350, p <0.001), and Hypothesis 1 is verified.

**Table 3:** Regression analysis of goal setting participation and employee active behavior.

Variable	PIS		EPB			
	M 1	M 2	M 3	M 4	M 5	M 6
**Gender**	-.008	-.011	-.034	-.037	-.030	-.033
**Age**	.012	.036	.238***	.261***	.232***	.248***
**Education**	.011	.058	-.031	.015	-.036	-.006
**Tenure**	-.018	-.023	-.069*	-.074*	-.061*	-.066*
**PGS**		.357***		.350***		.221***
**PIS**					.449***	.359***
**F**	.072	11.299***	6.290***	19.596***	28.272***	30.065***
**R2**	.001	.146	.071	.229	.300	.354
**Adust R2**	-.011	.133	.059	.217	.289	.342
**△R2**	.001	.145	.071	.158	.229	.125

Note

1) PGS; participation in goal setting, PIS; perceived insider status, EPB; active employee behavior

2) *** p<0.001, ** p<0.01, * p<0.05

This study used a hierarchical regression method to test the mediating effect of perceived insider status. The test results are shown in Table 3. Model 2 shows that goal-setting participation has a significant positive effect on perceived insider status (β = 0.357, p <0.001). Model 5 shows that employees’ perception of insider identity significantly impacts employees’ proactive behavior (β = 0.449, p <0.001). When the control variables, goal-setting participation, and perceived insider status were included in the regression equation (Model 6), The predictive effect of goal-setting participation on employee’s positive behavior weakened (β = 0.221, p <0.001), and perceived insider status significantly affected employee proactive behavior (β = 0.359, p <0.001). Perceived insider status plays a part of the mediating role between goal-setting participation and employee proactive behavior. The results obtained supported hypothesis 2.

Furthermore, the study in Table 4 conducted bootstrapping to test the mediation effect of insider status in the direct and indirect effect of goal-setting participation on employee practive behavior. Under the direct effect of goal-setting participation on employee proactive behavior. The direct effect is significant (95% confidence interval CI = [LLCI = 0.1385, ULCI = 0.3043] did not include 0). Hence the result supports Hypotheses 1. Under the condition, the indirect effect is significant (95% confidence interval CI = [LLCI = 0.853, ULCI = 0.1740], which did not include 0) and verifies hypothesis 2.

**Table 4:** Bootstrapping.

	Effect	se	LLCI	ULCI
**Direct effect of X on Y**	.2214	.0421	.1385	.3043
**Indirect effect(s) of X on Y**	.1283	.0229	.0853	.1740

### Test of moderating effects of power distance

In this study, the hierarchical regression method is used to test the moderating effect of power distance. The test results are shown in Table 5. Model 4 shows that goal-setting participation positively effects the perceived insider status (β = 0.260, p <0.001).An interactive item between goalsetting participation and power distance significantly affects perceived insider status (β = 0.027, p <0.05). It shows that both the coefficients are positive in nature and power distance positively moderates the relationship between goal-setting participation and perceived insider status, verifying Hypothesis 3.

**Table 5:** Regression results for testing moderating role of power distance.

Variable	PIS			
	M 1	M 2	M 3	M 4
**Gender**	-.008	-.011	.007	.012
**Age**	.012	.036	.030	.027
**Education**	.011	.058	.073	.073
**Tenure**	-.018	-.023	-.016	-.013
**PGS**		.357***	.259***	.260***
**PD**			.343***	.345***
PGS*PD				.027*
**F**	.072	11.299***	18.670***	19.074***
**R2**	.001	.146	.254	.265
**Adust R2**	-.011	.133	.240	.260
**△R2**	.001	.145	.108	.011

Note PGS; participation in goal setting, PIS; perceived insider status, EPB; active employee behavior, PD; power distance (2) *** p<0.001, ** p<0.01, * p<0.05

**Fig 2:**
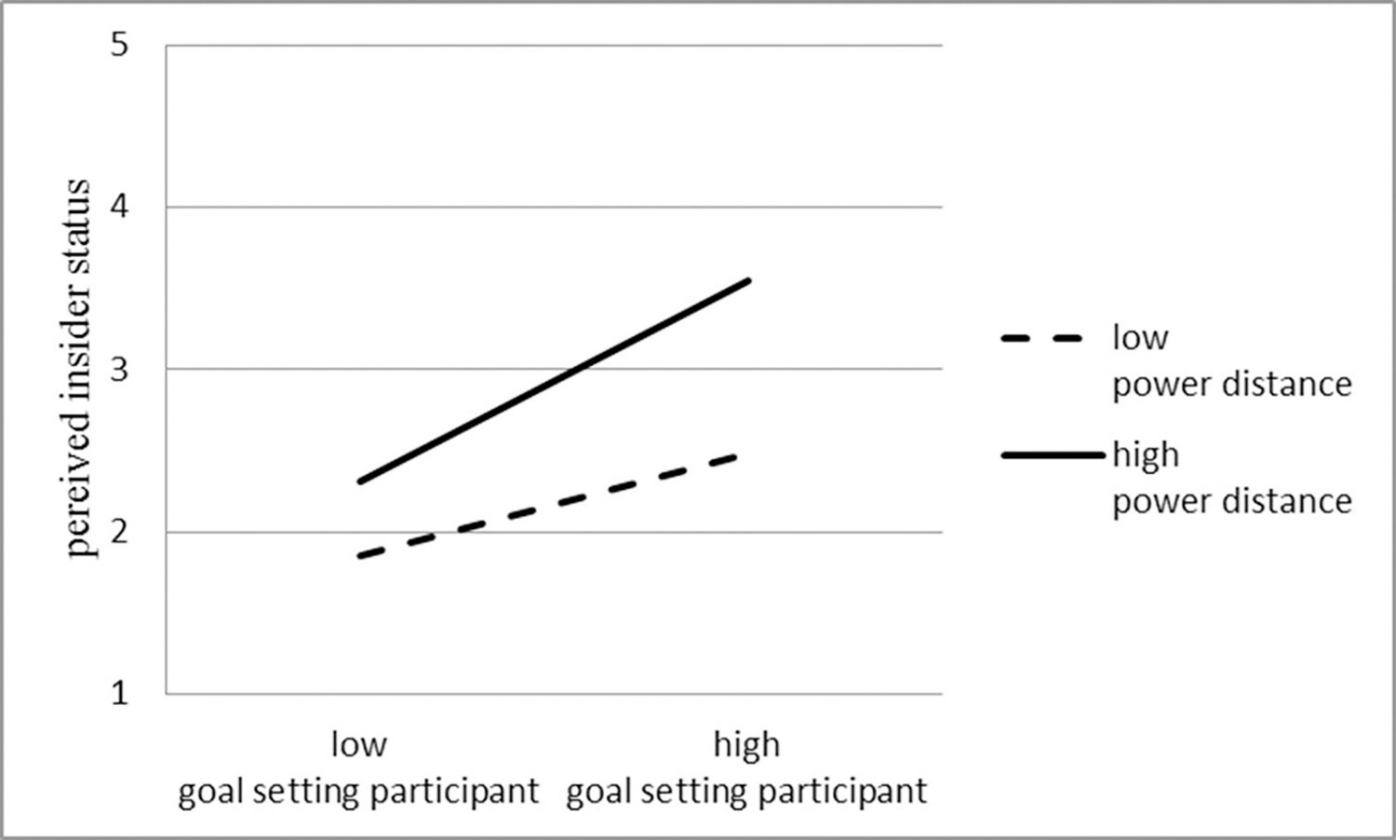
Power distance adjustment effect.

This study draws a graph of the power distance adjustment effect to describe further the direction and trend of the power distance adjustment effect, as shown in Fig 2. The test results are consistent with Hypothesis 3. The solid line represents the high power distance level group, and the dotted line represents the low power distance level group. The research results show when employees or organizations have a high level of power distance, the positive correlation between employees’ perception of insider identity and goal-setting participation is relatively steep. Whereas employees or organizations having a low power distance, The positive correlation between human identity perception and goal-setting participation is relatively flat. Therefore, Hypothesis 3 is verified.

### Test of moderated mediation

This research used Mplus to conduct the moderated mediation analysis, and the results are stated in Table 6. In low power distance, goal-setting participation on employee proactive behavior through perceived insider status is significant at 0.123***. In contrast, at high power distance, the effect of goal-setting participation on employee proactive behavior through perceived insider status is significant at 0.137***. The difference between the indirect effect of low power distance and high power distance is observed effective at 0.015*, proving the moderated mediation and supporting the model.

**Table 6:** Moderated mediation effect.

Moderator	PGS→PIS→EPB				
	Stage 1	Stage 2	Direct effect	Indrect effect	Total effect
**Low pd**	0.334***	0.347***	0.188**	0.123***	0.311***
**Highpd**	0.374***	0.387***	0.229***	0.137***	0.366***
**Difference**	0.040**	0.040	0.040*	0.015*	0.055

Note

*** p<0.001

** p<0.01

* p<0.05

## Discussion

This study built a theoretical model of the impact of goal-setting participation on employees’ proactive behavior centered on goal-setting theory and social cognitive theory. In this model, the perceived insider identity is used as an intermediary variable. The realization path of goal-setting participation to employee’s proactive behavior is also discussed. Furthermore, this study explores the positive regulating effect of goal-setting participation and perceived insider status using power distance as a moderator variable. It further influences the role of goal-setting participation in promoting employee’s proactive behavior. The questionnaire survey was used to verify the theoretical hypothesis. The research conclusions are as follows:

First, goal-setting participation has a positive impact on employees’ proactive behavior. When the organization’s degree of goal-setting participation is low, strengthening the organizational atmosphere of goal-setting participation can promote employees’ proactive behavior to a certain extent, consistent with the previous results [81, 82]. Based on goal-setting theory, the results proposed that goal-setting participation allows employees to clarify the accuracy and difficulty of goals, increases employees’ grasp of goals, and promotes employees’ proactive behavior. Clements & Kamau’s study seconds the idea of providing more autonomy in goal-setting to have more realistic and accurate goals [23].

Second, perceived insider status plays a mediating role in goal-setting participation and employees’ proactive behavior. The goal-setting and participation in the organizational context have given employees full respect, trust and strengthened their internal identity perception [83, 84]. Based on social cognition theory, employees develop a sense of “owner,” consistent with the research of Zagenczyk et al. [85] To consolidate employees’ status as “inners”, employees are more likely to have proactive behavior.

Third, power distance positively regulates the role of goal-setting participation on the perceived insider status and further affects employee’s proactive behavior. Results suggested and consistent with previous research that employees are unwilling to participate in goal-setting when an organization’s level of power distance is high [86]. Therefore, goal-setting participation is more robust, and a greater probability of employees’ proactive behavior. The specific performance is that in organizations with a low level of power distance, the organization’s managers believe that employee participation in decision-making can bring hitches to an organization and are keen to involve employees in the organization’s goal-setting less. Study results also suggest that employees with higher power distance have a higher acceptance of organizational inequality situations and have a lower need to set organizational goals [86]. Consistent with Anand et al.’s results, When the level of power distance is high, the lower the degree of employee participation in goal-setting, the stronger the perceived insider status [65]. The more significant the probability that the employee will take the initiative. Therefore, the level of power distance amplifies the effect of goal-setting participation.

In summary, goal-setting participation positively affects employee’s proactive behavior, and employee’s active behavior enhances organizational performance. Therefore, organizations should pay attention to and strive to create an organizational context for goal-setting participation.

### Practical implications

First of all, goal-setting participation is a positive work characteristic and should receive the organization’s attention. Goal-setting participation is an empowered and decentralized management method that can maximize the organization’s resources. In the organizational context of goal-setting participation, employees feel more autonomy, allowing employees to have a clearer understanding of the difficulty and accuracy of organizational goals. Employees can make comments and suggestions, increasing the degree of recognition of the organization’s goals and their achievability. Besides, goal-setting creates an atmosphere of mutual trust and respect between the organization and employees and enhances the perception of the identity of the insiders of employees. Therefore, the organization should create an atmosphere of employee participation in Goal-setting, encourage employees to participate more, and develop a reward system for employee suggestions.

Second, organizations should focus on how to improve employees’ perception of insider identity. Previous studies have considered the positive effect of perceived insider status on organizational development from different perspectives [87, 88]. When employees have a strong sense of insider identity, they will consider themselves and the organization a community of destiny. The organization’s development is associated with its development, resulting in positive behaviors conducive to its development. The stronger the employee’s perception of insider identity is, the greater the awareness of contributing to the organization. Therefore, organizations should continue to focus on how to increase employees’ internal identity awareness.

Finally, organizations should identify employees with different personality traits and adopt different management strategies. For example, employees with low power distances pursue an equal organizational climate. The organization should solicit more opinions from employees and be more willing to participate in goal-setting. Also, employees with low power distance levels should be selected to participate in the organization’s goal-setting in low-power distance organizations. This way, the employees’ maximum ability can maximize the organization and employees’ benefits. Organizations with different power distances should adopt different leaders and management methods. In organizations with a low level of power distance, empowered, participatory leaders can adopt effective management methods to promote proactive employee behavior.

## Research limitations and future research prospects

This study applies goal-setting theory and social cognition theory to explore the influence mechanism of goal-setting participation on employee’s proactive behavior and further verified it through empirical analysis. The study results confirm the positive effect of goal-setting participation on organizational development. It makes theoretical contributions to the academic community and provides practical suggestions for corporate growth. However, this research also inevitably has some limitations.

Although this study draws on previous developed foreign scales, the differences in Chinese and foreign cultures may still lead to situational unsuitability. Subsequent research can further explore localized scales to improve their practicality in China.

Secondly, The questionnaires used in this study are all self-evaluation questions. Some answers may not be objective and fair. Subsequent research may consider inquiring in combination with experimental methods to strengthen the research conclusions’ reliability.

Thirdly, Other control variables related to employee proactive behavior are not set except for the demographic information factors. Future research should consider more control variables like organizational support, personality and job satisfaction to better explain this relationship

Fourthly, The study’s conclusion was based on an examination of 20 firms of various types, some of which are state-owned, some of which are private, and some of which are joint ventures. Furthermore, these businesses were chosen because they span a wide range of industries, including education, banking, sales and services, and more. However, including additional firms would bring fresh perspectives to future studies.

Lastly, This study applies goal-setting theory and social cognition theory to explore the mediating role of perceived insider status and moderating role of power distance in the influence mechanism of goal-setting participation on employee’s proactive behavior. Previous studies have shown that organizational identity and other factors also positively affect employee’s proactive behavior. Therefore, subsequent studies can use other theories to explore other variables between goal-setting participation and employee’s active behavior mediating and moderating effect.
